# A Noise Reduction Method for Dual-Mass Micro-Electromechanical Gyroscopes Based on Sample Entropy Empirical Mode Decomposition and Time-Frequency Peak Filtering

**DOI:** 10.3390/s16060796

**Published:** 2016-05-31

**Authors:** Chong Shen, Jie Li, Xiaoming Zhang, Yunbo Shi, Jun Tang, Huiliang Cao, Jun Liu

**Affiliations:** National Key Laboratory for Electronic Measurement Technology, Key Laboratory of Instrumentation Science & Dynamic Measurement, Ministry of Education, School of Instrument and Electronics, North University of China, Taiyuan 030051, China; shenchong86@163.com (C.S.); lijie@nuc.edu.cn (J.L.); zxm_auto@nuc.edu.cn (X.Z.); shiyunbo@nuc.edu.cn (Y.S.); tangjun16@126.com (J.T.)

**Keywords:** dual-mass MEMS gyroscope, noise reduction, sample entropy, empirical mode decomposition, time-frequency peak filtering

## Abstract

The different noise components in a dual-mass micro-electromechanical system (MEMS) gyroscope structure is analyzed in this paper, including mechanical-thermal noise (MTN), electronic-thermal noise (ETN), flicker noise (FN) and Coriolis signal in-phase noise (IPN). The structure equivalent electronic model is established, and an improved white Gaussian noise reduction method for dual-mass MEMS gyroscopes is proposed which is based on sample entropy empirical mode decomposition (SEEMD) and time-frequency peak filtering (TFPF). There is a contradiction in TFPS, *i.e.*, selecting a short window length may lead to good preservation of signal amplitude but bad random noise reduction, whereas selecting a long window length may lead to serious attenuation of the signal amplitude but effective random noise reduction. In order to achieve a good tradeoff between valid signal amplitude preservation and random noise reduction, SEEMD is adopted to improve TFPF. Firstly, the original signal is decomposed into intrinsic mode functions (IMFs) by EMD, and the SE of each IMF is calculated in order to classify the numerous IMFs into three different components; then short window TFPF is employed for low frequency component of IMFs, and long window TFPF is employed for high frequency component of IMFs, and the noise component of IMFs is wiped off directly; at last the final signal is obtained after reconstruction. Rotation experimental and temperature experimental are carried out to verify the proposed SEEMD-TFPF algorithm, the verification and comparison results show that the de-noising performance of SEEMD-TFPF is better than that achievable with the traditional wavelet, Kalman filter and fixed window length TFPF methods.

## 1. Introduction

In the last decades, MEMS gyroscopes have been applied to inertial navigation, industrial control, consumer electronics, railway siding detection and other areas due to their advantages of low cost, small size and light weight [1,2]. During the application of a MEMS gyroscope, the acceleration along the sense axis causes great errors in the output signal, and the dual-mass gyroscope can prevent the mentioned phenomenon from occurring by employing differential detection technology. That’s the reason why dual-mass structures are attractive to numerous researchers [3,4]. However, the noise becomes an obvious bottleneck which limits the MEMS gyroscope performance. Therefore, the topic of dual-mass MEMS gyroscope noise elimination methods is studied in this work.

Many literatures have been dedicated great efforts to reduce MEMS gyroscope noise. References [5,6,7] indicated that the noise source of capacitive measurement-based MEMS structures mainly contains mechanical noise generated by the mechanical vibration of the polysilicon springs, the measurement system noise and the circuit electrical noise. Meanwhile, experiments have shown that the 1/*f* noise and the white Gaussian noise govern the low and high frequency domain, respectively. Normally, white Gaussian noise need to be reduced first, because the true signal and low frequency drift are usually submerged in the white Gaussian noise. Cao *et al.* proposed an improved differential interface and introduced a noise suppression method in sense open loop for a turning fork micro-gyroscope structure [8]; Xue *et al.* proposed a novel approach for processing the output signals of the MEMS gyroscope to reduce the bias drift and noise, where a Kalman filter was employed for filtering [9]; Yuan *et al.* proposed an improved noise reduction algorithm based on wavelet transformation for MEMS gyroscopes, and the experimental analysis results showed that the improved noise reduction method had good results in noise reduction of the gyroscope data at different frequencies [10]; Zhao *et al.* [11] proposed a new morphological wavelet filter algorithm which combined the advantages of wavelet filter and morphologic filter to process the output signals of MEMS gyroscopes, and the experimental results showed that the proposed morphological wavelet filter algorithm can reduce high frequency noises and suppress the random drift of MEMS gyroscopes effectively; Liu *et al.* [12] proposed a digital signal processing method for a micro-machined vibratory gyroscope based on a three dimensional adaptive filter demodulator which showed excellent performance in experimental verification. From the literature above it can be concluded that wavelet and Kalman filter are the most commonly used noise reduction method for MEMS gyroscopes. However, there are some other outstanding de-noising methods that have been proposed but never used for MEMS gyroscopes. For example, time-frequency peak filtering (TFPF) uses the instantaneous frequency estimation technique based on Wigner-Ville distribution (WWD) to recover signals corrupted by random noise, which has been successfully applied in seismic random noise reduction [13,14,15]. Obviously, TFPF is not a perfect de-noising method due to the fact the selection of TFPF window length significantly affects the signal preseration and noise attenuation performance. Numerous studies for window length selection of TFPF have been proposed, such as the Hurst exponent-based TFPF [16], SW statistic adaptive TFPF [17], curvature-varying hyperbolic trace TFPF [18], *et al.* which can effectively improve the performance of TFPF.

In this paper, the structure and noise analysis of dual-mass MEMS gyroscopes are introduced, and the application of TFPF for white Gaussian noise reduction of MEMS gyroscopes is investigated at the first time, then a novel design for window length selection of TFPF based on EMD and sample entropy is proposed, and at last, the experimental and comparison results are given.

## 2. Dual-Mass MEMS Gyroscope and Noise Analysis

### 2.1. Dual-Mass MEMS Gyroscope

**Figure 1:**
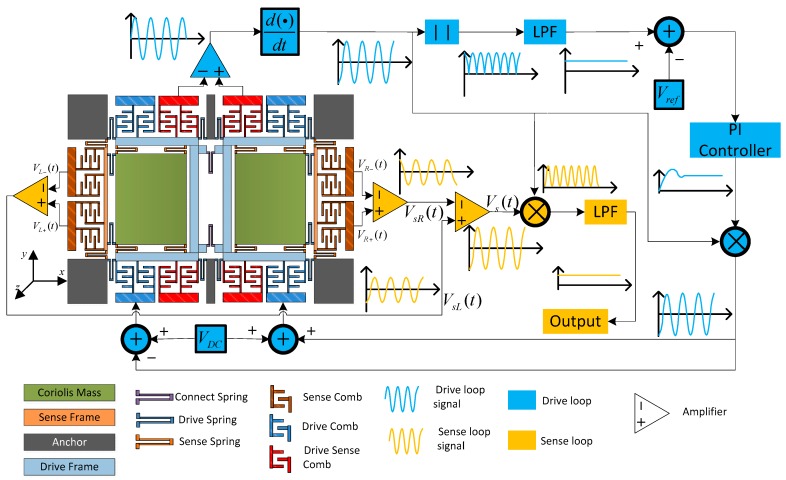
Schematic of the dual-mass decoupled gyroscope structure and periphery circuit.

Sense axial accelerations often cause the invalidation of the sense signal in single mass MEMS gyroscope structures, so a dual-mass structure method is proposed to suppress this phenomenon. An AGC loop can stabilize the drive mode vibrating amplitude based on self-oscillation theory, and the method ensures the drive mode works at its own resonant frequency. In this case, gyroscope mechanical sensitivity achieves the maximum values with same *A_x_*. The sense loop utilizes open-loop method and phase sensitive demodulation technology, and a schematic of the gyroscope structure and peripheral circuits is shown in Figure 1 [8].

**Figure 2:**
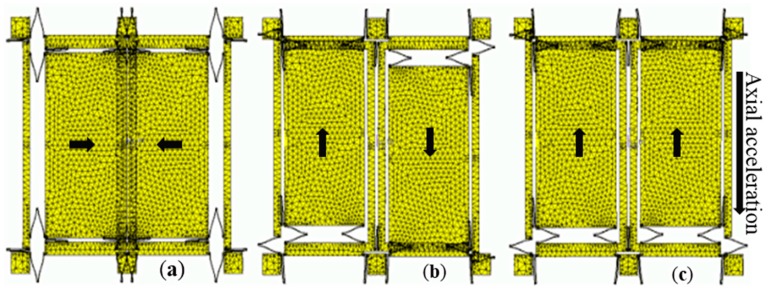
Working modes of the gyroscope. (**a**) Drive mode; (**b**) Sense mode with Coriolis force; (**c**) Sense mode with axial acceleration.

The structure working principle of a dual-mass structure is introduced in Figure 2: drive frames of two masses are stimulated along inverse orientations with the same amplitude (Figure 2a), so *Ω_z_* generates inverse displacements (Coriolis signal) upon two sense frames (Figure 2b). The sense axial acceleration generates two sense frames in-phase displacements (Figure 2c), which can be regarded as the common mode error. This structure is ceramic vacuum packaged [19]. The differential detection interface is employed to extract the Coriolis signal and decrease the in-phase component and common mode error.

**Figure 3:**
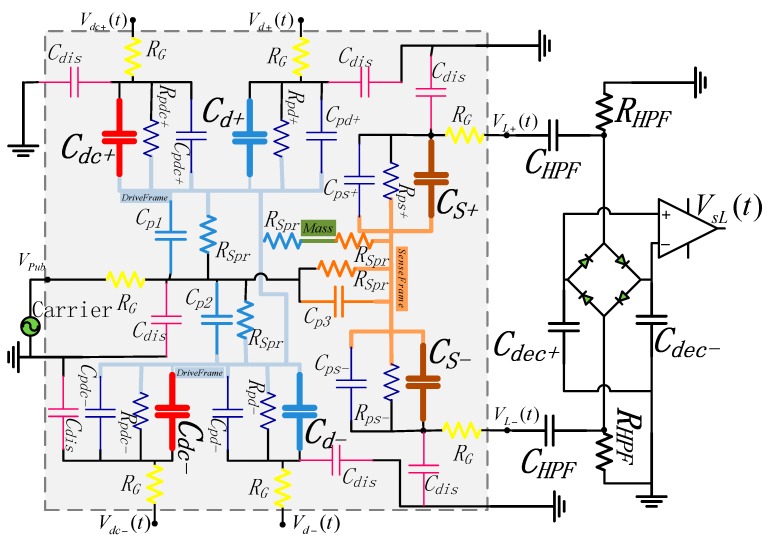
Electric model and interface of each single mass.

The left mass (same with the right mass) equivalent electric model and the interface circuit are shown in Figure 3 [8]. In this schematic, the structure of the electronic model is in the gray dashed box, and several electronic nodes and elements are stressed with the corresponding color in Figure 1: Drive Frame, Sense Frame, Coriolis Mass, the resistors of the springs (*R_Spr_*, can be calculated and is about several hundred ohm), Sense Capacitances (*C_s_+*, *C_s_−*), Drive Capacitances (*C_d_+*, *C_d_−*) and Drive Sense Capacitances (*C_dc_+*, *C_dc_−*). The design of capacitances are over striking and the design values are about several pF. The dark blue ones are the parasitic capacitors and resistors (R_p*_, several hundred MΩ, can be measured by a multimeter) between silicon structure and the metal film on the glass basement. The metal lines produce the distributed capacitors, which are the pink ones (*C_dis_*). The resistors marked with “*R_G_*” (yellow ones, which can be measured by a multimeter, and are about several ohm) are the resistors of gold lines connecting the package pins. The interface includes a diode bridge, two precise detection capacitances (*C_dec_+*, *C_dec_−*), two high pass filter (formed by *C_HPF_* and *R_HPF_*) and an instrument amplifier, the carrier frequency *f_c_* is about several MHz.

It can be obtained from various references that the most noise components focus on the low frequency domain, and the Coriolis signal is extremely weak and may be submerged in the noise signal, so it is necessary to separate the useful signal from the noise background so as to improve the Coriolis signal noise ratio (SNR).

### 2.2. Noise Analysis

From the previous research, it can be concluded that there are several typical noises in bulk micro-machined capacitance detection devices, and the sorts of noise in this paper are introduced in following five parts [8]:

(1) Mechanical-Thermal Noise (MTN). This noise exists widely in MEMS gyro structures, and is caused by molecular agitation inside the silicon structure; it is commonly considered as the mechanical sensitivity limitation. The essence of MTN is white Gaussian noise and can be equal to adding a noise force generator together with damper, which can be expressed as:
  (1) $$F_{MTN}=\sqrt{4k_{B}TcB}$$
  where *k_B_* is the Boltzman constant, *T* is the absolute temperature, *c* is the gyro mode damping, and *B* is the noise bandwidth.
(2) Electronic Thermal Noise (ETN). This noise is also named Johnson noise, which is caused by charge thermal agitation in resistors. The voltage generated by ETN can be described as:
  (2) $$V_{ETN}=\sqrt{4k_{B}TRB}$$
  where *R* is the resistance. Although ETN exists in both the structure electronic model and the periphery circuit, ETN (mainly produced by the parasitic resistors) considered in this paper causes more negative influences because the weak signal produced by sense capacitors is more vulnerable to ETN. From Figure 1, it can be found that the distributed resistances *R_pdc_+*, *R_pdc_−*, *R_ps_+* and *R_ps_−* are high-value ones (their values are several hundred MΩ), therefore, the sense loop output signals will be easily influenced by their ETN.
(3) Flicker Noise (FN). This noise is well known as 1/*f* noise and generated by the result of conductivity fluctuations in a semiconductor device, This paper expresses its equivalent voltage as:
  (3) $$V_{FN}=\sqrt{KR^{2}\frac{I}{f}B}$$
  where *K* is a constant that depends on the type of material and its geometry, *R* is the resistor value, *I* is the average value of direct current, *f* is the frequency of the passing signal. From the equation above, we can find that FN doesn’t depend on the temperature, and it is inversely proportional to *f*, so increasing carrier frequency *f_c_* is an effective way to restrain FN in a silicon structure.
(4) In Phase Noise (IPN). This kind of noise has the same frequency and phase characteristics as the Coriolis signal in the sense loop. Normally it contains the cross-coupling of the drive signal (through drive and sense capacitance) and the sense mode motion directly stimulated by drive signals (caused by the non-idealities in the structure element), so different gyroscopes have different IPN. The IPN in this paper can be described as:
  (4) $$V_{IPN}(t)=A_{IPN}\cos\omega_{d}t$$
  where *A_IPN_* is the amplitude of IPN equivalent voltage. Usually, its equivalent input angular rate is several deg/s and it cannot be eliminated.
(5) Other Noise (OTN). The above four noises sources form the principal parts of structure noise, besides which, there are also several noises existing in MEMS gyroscope, such as diode noise, demodulation phase noise, residual stress noise and so on.

## 3. Noise Reduction Algorithm

### 3.1. Sample Entropy Based Empirical Mode Decomposition

There are two key parts in the EMD technique, which are instrinsic-mode functions (IMFs) and the sifting process. An IMF is a function which satisfies two conditions [20,21]:

(1) The number of extrema and the number of zero crossings should be equal or maintain a difference of no more than one;
(2) The local average defined by the average of the maximum and minimum envelops is zero, *i.e.*, both envelopes are locally symmetric around the envelope mean.

Briefly speaking, the EMD method involves determining the instantaneous equilibrium position based on time series, the average of the upper and lower envelope. Then the non-stationary signal is decomposed into a set of linear and stationary IMFs. Given a signal x(t), the first step of EMD is the identification of all the local maxima and minima. Then all the local maxima are connected by a cubic spline curve as the upper envelope $e_{u}\left(t\right)$. Similarly, all the local minima are connected by a spline curve as the lower envelope $e_{l}\left(t\right)$. The mean value of the two envelopes is denoted as $m_{l}\left(t\right)=\left[e_{u}\left(t\right)+e_{l}\left(t\right)\right]/2$ and is subtracted from the original signal. Thus, the first proto-IMF $h_{l}\left(t\right)$ is obtained:

(5) $$h_{l}\left(t\right)=x\left(t\right)-m_{l}\left(t\right)$$

The procedure above for extracting the IMFs is referred to as the sifting process. Since $h_{l}\left(t\right)$ still contains multiple extrema between zero crossing, the sifting process is performed again on $h_{l}\left(t\right)$. This process is applied repetitively to the proto-IMF $h_{k}\left(t\right)$ until the first IMF $c_{l}\left(t\right)$ is obtained, where the $c_{l}\left(t\right)$ satisfies the IMF conditions. The stopping criteria is employed to terminate the sifting process where the sum of difference (SD) is used:

(6) $$\text{SD}=\frac{\sum_{t=0}^{T}{\left|h_{k-1}\left(t\right)-h_{k}\left(t\right)\right|}^{2}}{\sum_{t=0}^{T}h_{k-1}{}^{2}}$$

When the SD is smaller than a threshold, the first IMF $c_{l}\left(t\right)$ is obtained, which is written as:

(7) $$x\left(t\right)-c_{1}\left(t\right)=r_{1}\left(t\right)$$

Note that the residual $r_{1}\left(t\right)$ still consists some useful information. Therefore the residual $r_{1}\left(t\right)$ is considered as a new signal and the procedure above is applied again, then the equation below is obtained:

(8) $$r_{i-1}\left(t\right)-c_{i}\left(t\right)=r_{i}\left(t\right)\;i=1,\ldots N$$

The whole procedure would be terminated when the residual $r_{N}\left(t\right)$ is either a constant, a monotonic slope, or a function with only one extremum. The Equations (7) and (8) are combined, then the Equation (9) is obtained:

(9) $$x\left(t\right)=\overset{N}{\underset{n=1}{\sum }}c_{n}\left(t\right)+r_{N}\left(t\right)$$

From Equation (9) it can be seen that the result of the EMD produces N IMFs and a residual signal. For convenience, we refer to $c_{N}\left(t\right)$ as the *n*th-order IMF. In this convention, lower-order IMFs reflect high frequency white Gaussian noise, and high-order IMFs reflect low frequency drift and true signals.

After decomposition by EMD, the original gyroscope’s signal will be decomposed into numerous IMFs. Actually, in this paper, the temperature error of gyroscope is expected to be decomposed into true signal components, mixed components (true signal and noise) and noise components. If we can classify the numerous IMFs into the three kinds of components above, the noise reduction process will be more clearly and specifically. Therefore, the sample entropy (SE) is introduced. SE examines time series for similar epochs and assigns a non-negative number to the sequence, with larger values corresponding to more complexity or irregularity in the data. SE(*m*, *r*, *N*) can be obtained by specifying a run length *m* and a tolerance window *r*. The detailed introduction of SE can be found at [22]:

(10) $$SE(m,r)=\underset{N\to \infty }{lim}\left\{-\ln\left[\frac{A^{m}r}{B^{m}r}\right]\right\}$$

### 3.2. Time-Frequency Peak Filtering

Conventionally, MEMS gyroscope signals contain true signal, drift and white Gaussian noise. In our study, the target is to reduce the white Gaussian noise. Therefore the Equation (11) is obtained:

(11) y(n)=x(n)+r(n)

where, x(n) contains true signal and drift; r(n) is the white Gaussian noise, and n is the sampling point. The noisy signal y(n) is encoded by frequency modulation as instantaneous frequency of unit amplitude analytic signal so as to actualize TFPF, which can be defined as Equation (12) in this paper:

(12) $$z_{y}\left(n\right)=e^{j2\pi \rho \sum_{m=0}^{n}y\left(m\right)}$$

where, ρ is a scaling parameter analogous to the frequency modulation index. After frequency modulation and encoding, the noisy signal y(n) converted into the instantaneous frequency of the analytic signal $z_{y}\left(n\right)$.

Then, the recovered signal can be obtained as Equation (13) by estimating the peak in the frequency of pseudo Wigner–Ville distribution of $z_{y}\left(n\right)$:

(13) $${\overset{\wedge }{x}}_{h}\left(n\right)=f_{z}\left(n\right)=\frac{\underset{f}{\arg\max\left[PW_{z}\left(n,f\right)\right]}}{\rho }$$

where, $PW_{z}\left(n,\;f\right)$ represents the pseudo Wigner–Ville distribution of $z_{y}\left(n\right)$ and the pseudo Wigner–Ville distribution with time-varying window *h*(*m*) is defined as follows:

(14) $$PW_{z}\left(n,f\right)=\sum_{m=-\infty }^{\infty }h\left(m\right)\bar{z_{y}}\left(n-m\right)z_{y}\left(n+m\right)e^{-j4\pi fm}$$

where, $\bar{z_{y}}$ is the conjugate operator to $z_{y}$. The length of window function *h*(*m*) is a trade-off parameter for random noise attenuation and signal preservation.

### 3.3. Steps of SEEMD-TFPF Algorithm

**Figure 4:**
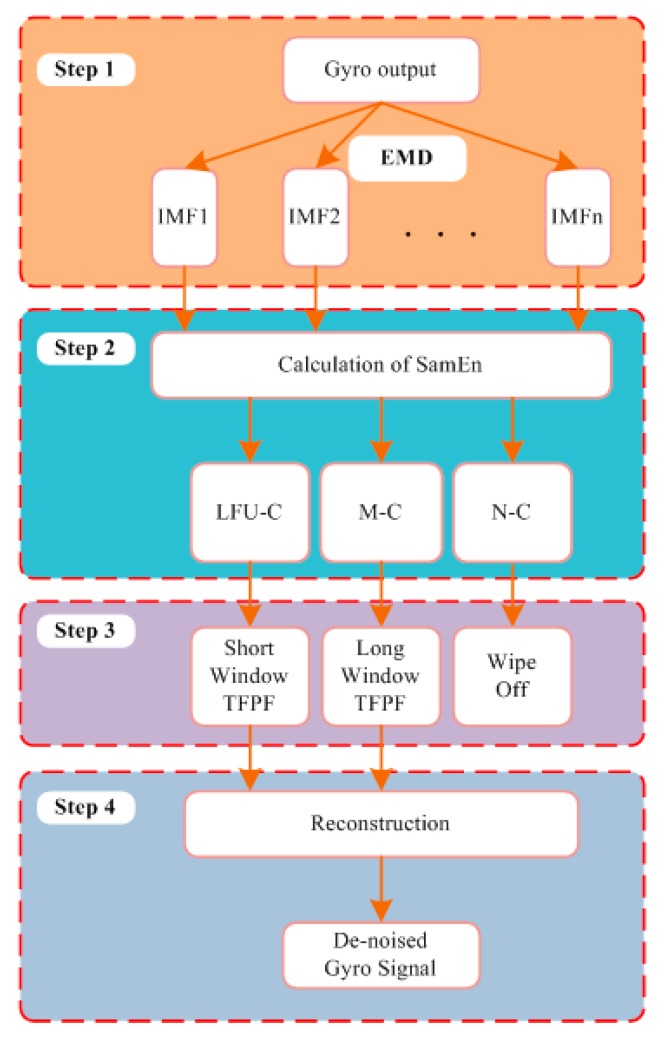
Steps of the proposed SEEMD-TFPF algorithm.

In order to combine the advantages of EMD and TFPF, the hybrid noise reduction algorithm SEEMD-TFPF is proposed. There are four steps of the proposed algorithm as shown in Figure 4.

*Step 1:*  *Decomposition*. The original signal is decomposed into IMFs by EMD;
*Step 2:*  *Classification*. The SE of each IMF is calculated, and then the IMFs with similar SE value are classified into one component. By analyzing the gyroscope data, normally three components can be obtained according to the similarity of SE, which are named as low frequency useful component (LFU-C), mixed component (M-C) and noise component (N-C), respectively.
*Step 3:*  *De-noising*. From Step 2 we can see that the LFU-C might consisted of the true signal and drift, the M-C might consisted of true signal, drift and noise, and the N-C consists of noise. It is necessary to choose different de-noising methods for the three different components. Therefore, considering the features of TFPF, short window TFPF is selected to process LFU-C in order to preserve the valid signal as much as possible, and long window TFPF is selected to process M-C in order to reduce the random noise as much as possible, at last the N-C would be wipe off directly.
*Step 4:*  *Reconstruction*. After de-noising, the LFU-C and M-C are reconstructed, and the final signals are obtained.

## 4. Experimental and Results

### 4.1. Rotation Experimental and Comparison

**Figure 5:**
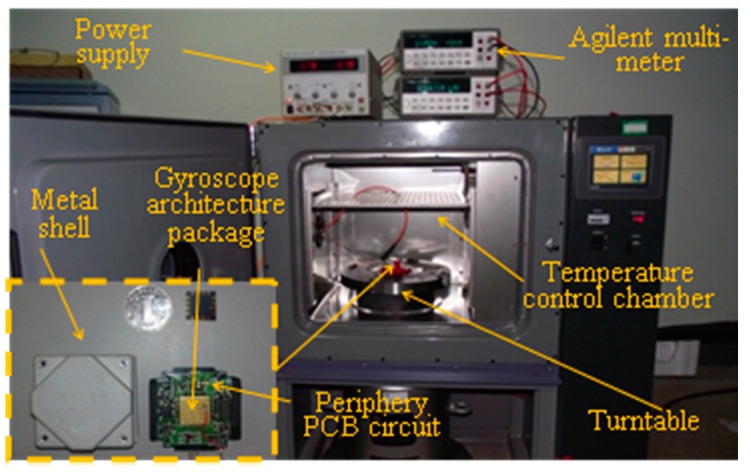
Test equipments for dual mass MEMS gyroscope.

In order to test the proposed SEEMD-TFPF noise reduction algorithm, a gyroscope output collection experiment is carried out. The gyroscope is placed in a temperature controlled oven as shown in Figure 5. The gyroscope output data is collected by an Agilent 34401A Digital Multi-meter and 1000 points are collected per hour, while the power is supplied by an Agilent E3631A DC Power Supply. Firstly the rotation experiment is carried out. The input rotation rates of rotary table are set as −1 °/s, −0.8 °/s, −0.6 °/s, −0.4 °/s, −0.2 °/s, 0.2 °/s, 0.4 °/s, 0.6 °/s, 0.8 °/s, 1 °/s. The sampling time of each rotation rate is 200 s.

**Figure 6:**
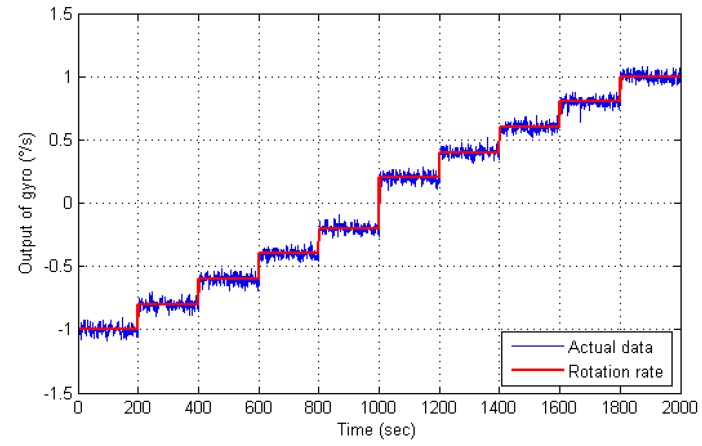
Rotation experimental results of a dual-mass MEMS gyroscope.

Figure 6 is the rotation experimental results. It can be clearly seen that the true signals are submerged in the white Gaussian noise, which would decrease the performance of a dual-mass MEMS gyroscope. Therefore, it is important to reduce the white Gaussian noise.

**Figure 7:**
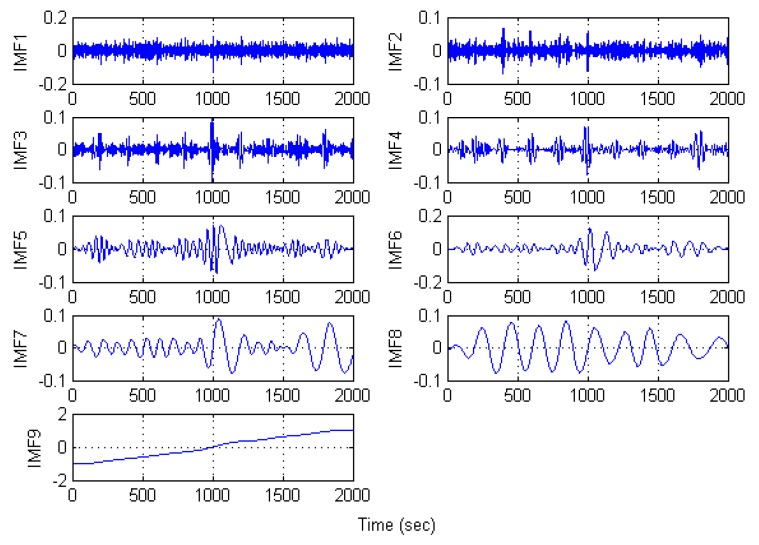
IMFs obtained by the rotation signal decomposition of EMD.

**Figure 8:**
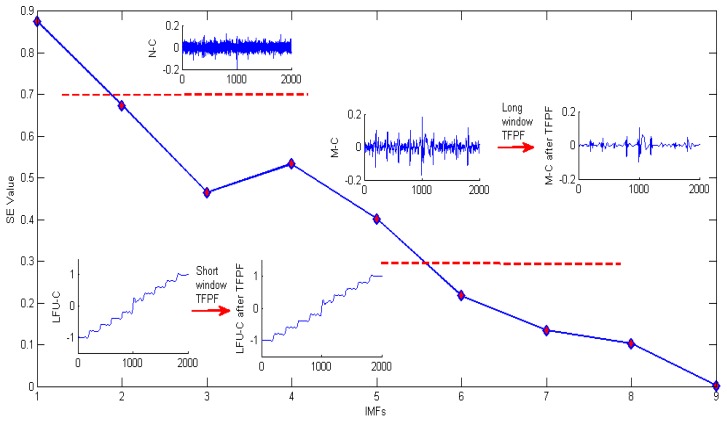
Detailed de-noising process of SEEMD-TFPF for rotation signal.

Figure 7 is the IMFs obtained after EMD decomposition and there are nine IMSs in total. According to the hybrid de-noising algorithm proposed by reference [23], all the IMFs would be processed by filters, which means nine filters are needed in our study. Obviously, the workload is too big if nine filters are employed. Therefore, it is necessary to simplify the filtration process. According to the proposed SEEMD-TFPF algorithm, the sample entropy of each IMF is calculated and the IMFs with similar value of sample entropy will be reconstructed. Figure 8 shows the process in detail.

**Figure 9:**
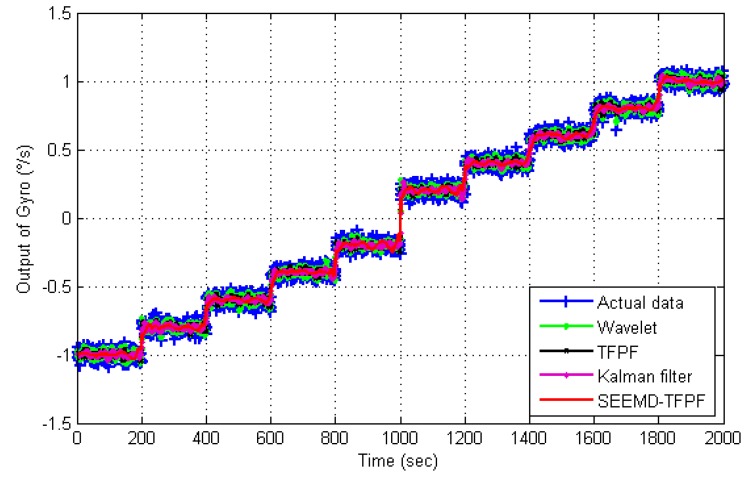
Comparison results of rotation signal de-noising by using different methods.

From Figure 8 it can be clearly seen that according to the value of sample entropy, the IMFs are classified into three components which are named low frequency useful component (true signal), mixed component (true signal and noise) and noise component (white Gaussian noise). Then according to the characteristics of TFPF, long window TFPF is selected to filter the mixed component (M-C) in order to reduce the noise as much as possible, and short window TFPF is selected to filter the low frequency useful component (LFU-C) in order to preserve the useful signal. As shown in Figure 8, the noise component is eliminated directly. Finally, the filtered M-C and LFU-C are reconstructed to obtain the final signal as shown in Figure 9.

Figure 9 is the de-noising results using SEEMD-TFPF and the comparison between different de-noising algorithms. Traditional wavelet algorithm and TFPF with fixed window length are employed for comparison. From Figure 9 it can be clearly seen that the proposed SEEMD-TFPF has the best de-noising ability. The standard deviations of each de-noising result are calculated and compared in Table 1, and the comparison results verify that the proposed SEEMD-TFPF has better de-noising performance than the traditional wavelet and TFPF methods.

### 4.2. Temperature Experimental and Comparison

**Figure 10:**
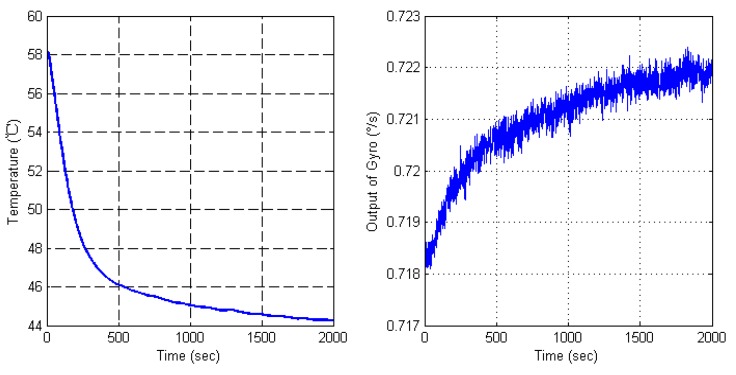
Temperature experimental results of a dual-mass MEMS gyroscope.

Another set of MEMS gyroscope signals which was collected in the temperature oven is utilized to verify the SEEMD-TFPF algorithm. A thermal resistance is employed to get the real time temperature inside the gyroscope metal shell, which value is synchronous with gyro output. The gyro is fixed on the static turntable to make sure the output signal is not influenced by any movement, two thermal resistance output lines are connected to another Agilent 34401A Digital Multi-meter to pick up thermal resistance values. The gyro power and output cable lines connect with instruments outside the oven. The gyro is powered on for one hour under room temperature, then, the oven is warmed up to 58 °C at high speed. The oven temperature is kept at 58 °C for one hour to make sure the temperature inside the gyro shell is 58 °C, and the thermal resistance value proves that. Then the data collection process begins, the temperature is decreased from 58 °C to 45 °C, and the temperature of oven is set to stop at 45 °C for 2000 s. This process makes sure the gyroscope inside-shell temperature is stable and equal to the oven temperature. The temperature variation and data collection results are shown in Figure 10. It can be clearly seen that there is an obvious drift trend. However, the drift is submerged into the white Gaussian noise, which cannot be compensated directly. Therefore, it is important to reduce the white Gaussian noise.

**Figure 11:**
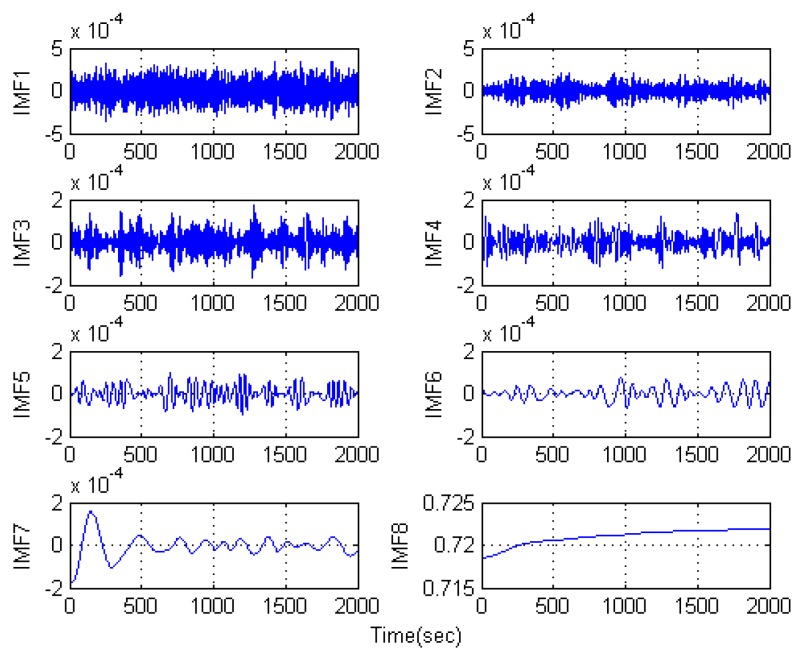
IMFs obtained by the temperature drift signal decomposition of EMD.

**Figure 12:**
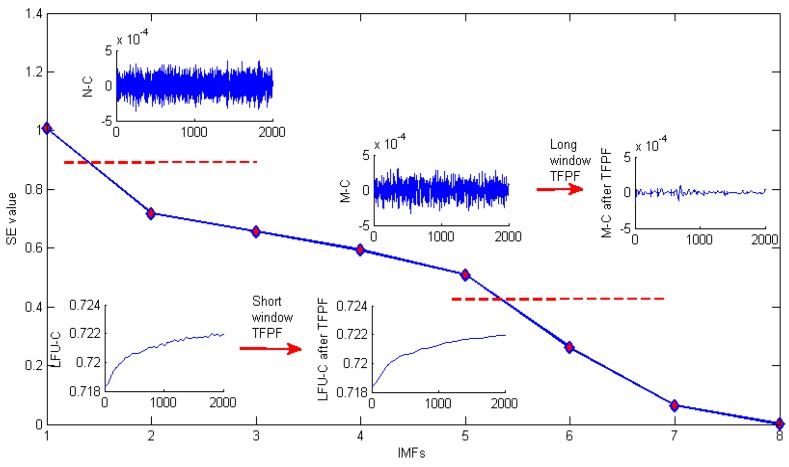
Detailed de-noising process of SEEMD-TFPF for temperature drift signal.

Figure 11 is the IMFs obtained after EMD decomposition and there are eight IMSs in total. According to the proposed SEEMD-TFPF algorithm, the sample entropy of each IMF is calculated and the IMFs with similar value of sample entropy will be reconstructed. Figure 12 shows the detailed process.

**Figure 13:**
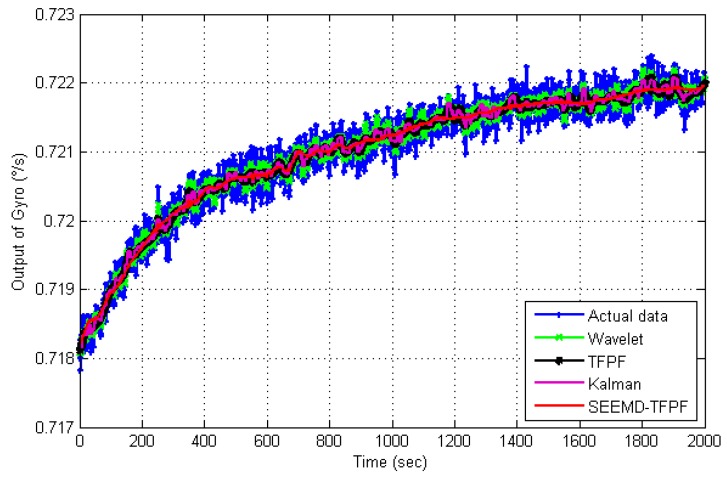
Comparison results of temperature drift signal de-noising by using different methods.

From Figure 12 it can be clearly seen that the low frequency useful component (drift), mixed component (drift and noise) and noise component (white Gaussian noise) are obtained. Then a long window TFPF is selected to filter the M-C in order to reduce the noise as much as possible, and a short window TFPF is selected to filter the LFU-C in order to preserve the drift. As shown in Figure 12, the noise component is eliminated directly. Finally, the filtered M-C and LFU-C are reconstructed to obtain the final signal as shown in Figure 13.

Figure 13 is the de-noising results by using SEEMD-TFPF and the comparison between different de-noising algorithms. Like in Figure 9, the traditional wavelet algorithm, Kalman filter and TFPF with fixed window length are employed for comparison. From Figure 13 it can be clearly seen that the proposed SEEMD-TFPF has the best de-noising ability. Standard deviations of each de-noising result are calculated and compared in Table 2, and the comparison results verify that the proposed SEEMD-TFPF has better de-noising performance than the traditional wavelet, Kalman filter and TFPF.

## 5. Conclusions

This paper investigated the noise elements in dual-mass MEMS gyroscopes, including mechanical-thermal noise, electronic-thermal noise, flicker noise and Coriolis signal in-phase noise. Then a noise reduction algorithm based on SEEMD and TFPF is proposed. The proposed SEEMD-TFPF method is different to prior de-noising methods, firstly the TFPF is introduced to process the MEMS gyroscope’s output signal for the first time, secondly SEEMD is employed to combine with TFPF in order to improve the performance TFPF.

Rotation experiments and temperature experiments are carried out, and the outputs are collected to verify the effectiveness of the proposed algorithm. Experimental and comparison results show that no matter whether in rotation or experimental circumstances, the proposed SEEMD-TFPF has the best de-noising ability compared to the traditional wavelet algorithm and fixed window length TFPF, and the white Gaussian noise of dual-mass MEMS gyroscope can be effectively suppressed.

## Figures and Tables

**Table 1:** SD comparison of different de-noising methods for rotation signal.

De-Noising Methods	Actual Data	Wavelet	TFPF	Kalman	SEEMD-TFPF
Standard deviation (°/s)	0.7129	0.6637	0.6018	0.4539	0.2634

**Table 2:** SD comparison of different de-noising methods for temperature drift signal.

De-Noising Methods	Actual Data	Wavelet	TFPF	Kalman	SEEMD-TFPF
Standard deviation (°/s)	9.74E−4	9.03E−4	8.89E−4	7.18E−4	4.54E−4
